# Three-Dimensional-Printed Osteochondral Scaffold with Biomimetic Surface Curvature for Osteochondral Regeneration

**DOI:** 10.3390/pharmaceutics17020153

**Published:** 2025-01-23

**Authors:** Yan Yang, Qu Lin, Zhenhai Hou, Gensheng Yang, Lian Shen

**Affiliations:** 1 College of Pharmaceutical Science, Zhejiang University of Technology, Hangzhou 310014, China; yangyan10@zjut.edu.cn (Y.Y.); 211122070116@zjut.edu.cn (Q.L.); 2 Department of Orthopaedics, No. 903 Hospital of PLA Joint Logistic Support Force, Hangzhou 310013, China; hou310000@126.com; 3 XiangFu Laboratory, Jiaxing 314102, China

**Keywords:** 3D printing, dimensional accuracy, biomimetic osteochondral scaffold, curvature distribution, osteochondral regeneration

## Abstract

Objectives: Treatment of osteochondral defects is hindered by several challenges, including the failure of traditional scaffolds with a predefined cylindrical or cuboid shape to comprehensively match the natural osteochondral tissue. Herein, we employed reverse modeling and three-dimensional (3D) printing technologies to prepare subchondral bone and cartilage. Methods: The osteochondral scaffold was prepared by bonding the subchondral bone and cartilage layers, and the curvature distribution and biomechanical behavior were compared with those of the native tissue. Biocompatibility and osteochondral regeneration performance were further evaluated using cell adhesion and proliferation assays, as well as animal osteochondral defect repair tests. Results: We found that increasing the printing temperature or decreasing the layer height improved the dimensional accuracy of printed subchondral bones, whereas increasing the exposure time or decreasing the layer height enhanced the dimensional accuracy of the printed cartilage. Biomimetic scaffolds exhibited curvature distribution and biomechanical behavior more similar to native tissues than traditional cylindrical scaffolds. Incorporating gelatin methacryloyl into poly (ethylene glycol) diacrylate markedly improved the biocompatibility, and correspondingly prepared osteochondral scaffolds had better osteochondral regeneration ability than the traditional scaffolds. Conclusions: Osteochondral scaffolds exhibiting biomimetic morphology and an internal structure could be prepared based on reverse modeling and 3D printing, facilitating personalized osteochondral injury treatment.

## 1. Introduction

Osteochondral defects, mainly caused by traumatic injuries and osteoarthritis [1], adversely affect global health and millions of individuals worldwide [2]. The treatment of osteochondral defects remains challenging owing to the distinct mechanical properties and physiological characteristics of cartilage and subchondral bones [3]. Different osteochondral scaffolds have been developed to mimic the mechanical properties of native tissues and to reconstruct defective osteochondral tissues. For example, a bilayer osteochondral scaffold comprising layer-specific stem cell differentiation inducers was developed to achieve the simultaneous regeneration of osteochondral tissues [4]. Additionally, a triphasic scaffold was successfully fabricated to synergistically enhance chondrogenesis and osteogenesis, thus serving as a tool for osteochondral pathology and drug screening [5]. Moreover, gradient scaffolds were found to better mimic the smooth transition of structure and composition between the cartilage and subchondral bone [6], thereby effectively circumventing poor integration at the interfaces of multilayered scaffolds [7].

Three-dimensional (3D) printing is an additive manufacturing technique that employs layer-by-layer deposition for fabrication [8]. Tissue-engineered scaffolds are frequently manufactured using 3D printing techniques to achieve structural complexity for precise and personalized therapy [1]. For example, fused deposition modeling (FDM) and digital light processing (DLP) have been successfully employed to fabricate hybrid bilayer scaffolds capable of promoting in vivo cartilage and subchondral bone repair and regeneration [9]. In addition to multilayer and gradient adjustments, 3D-printed scaffolds can be customized for cell attachment and guidance. For example, integrated osteochondral scaffolds with biomimetic hierarchical structures and distinct channels have been fabricated using a selective laser sintering technique, which could greatly advance osteochondral regeneration without cells or growth factors [10]. Given that general osteochondral scaffolds (i.e., cylinders and cuboids) fail to comprehensively match the surrounding natural tissue, a customized biomimetic scaffold based on 3D printing could be considered.

However, biomimetic osteochondral scaffolds exhibit complex and irregular morphologies, which are difficult to model using conventional software. In recent years, reverse modeling techniques, such as 3D scanning combined with 3D printing, have been employed in the fields of stomatology and dermatology. Notably, 3D scanning technology was applied to obtain a 3D model of the nose, and flexible, personalized drug-loaded devices have been prepared using 3D printing technologies to treat acne [11]. For personalized treatment, 3D scanning combined with 3D printing technology can achieve the biomimetic morphology and internal structure of osteochondral scaffolds.

Dimensional measurement is a testing consideration for scaffolds, as stated in the US Food and Drug Administration guidance for additive-manufactured medical devices [12]. Dimensional accuracy is one indicator used to evaluate the morphology of medical devices. Three-dimensional-printed dental devices with high dimensional accuracy have been widely studied. Three-dimensional printing technologies, printing parameters, support parameters, and post-processing procedures can impact the accuracy of 3D-printed dental devices [13]. For example, 3D printing techniques and build angles substantially impact the dimensional accuracy of 3D-printed palatal plate orthodontic appliances [14]. The accuracy of dental diagnostic models is also influenced by base-cast shapes, among which the hollowed base cast affords the highest accuracy [15]. Furthermore, the shape similarity level was another indicator used to evaluate the morphology of biomimetic scaffolds. For example, an ear-shaped scaffold can be fabricated by pressing the material into a negative mold. According to the shape analysis, the similarity level of the scaffold, compared with that of the normal ear digital mold, exceeded 97% [16]. Human ear- and nose-shaped scaffolds were successfully fabricated using photocuring 3D printing, with scaffold shape similarity levels exceeding 90% [17]. For osteochondral scaffolds with a biomimetic morphology, the dimensional accuracy of scaffolds is crucial for ensuring the effectiveness and safety of personalized treatment.

In addition to dimensional accuracy and model matching, the biomechanical behavior of biomimetic implants is critical. Finite element analysis (FEA) is an ideal tool for evaluating the biomechanical behavior of the knee joint, as it not only helps reduce treatment costs but can also aid in enhancing methodologies and developing new ones [18]. FEA has been used to analyze cartilage defects and subsequent tissue loading, simulate stress and strain distributions, and predict regions where mechanical failure is more likely to occur during osteochondral defect progression [19]. Furthermore, FEA has been used to assess the outcomes of anterior cruciate ligament reconstruction techniques and stresses and strains in the tibial cartilage during the stance phase [20]. Previously, we simulated the biomechanical behavior of the proximal tibia and added an elastic layer to mimic cartilage and articular cavities. The biomimetic orthopedic implants had good compression resistance, with the articular cavity and cartilage affording cushioning effects [21]. Given that animals experience physiological changes more rapidly [22], biomimetic simulations of animal (i.e., rabbit) knee joints using FEA can improve our understanding of the biomechanical behavior of knee joints.

Multilayer structures of the osteochondral scaffold could accurately mimic the complexity of the osteochondral tissue to facilitate the formation of new tissue. Biomimetic morphology matches the natural osteochondral tissue and also enhances the formation of new tissue. In the current study, we fabricated subchondral bone and cartilage using 3D scanning combined with 3D printing technologies. The printing parameters were optimized based on dimensional accuracy to achieve biomimetic morphology. For a biomimetic internal structure, the internal grid and printing material were optimized based on their mechanical properties. A tensile test was performed to examine the bonding strength of the biomimetic osteochondral scaffolds with different interfacial curvatures. Curvature distribution and biomechanical behavior of the biomimetic scaffold were compared with those of native tissues. Finally, the biocompatibility and osteochondral regeneration abilities of osteochondral scaffolds were determined both in vitro and in vivo.

## 2. Materials and Methods

### 2.1. Materials

Polylactic acid filaments (PolyLite^TM^ PLA; No. 200708109) were purchased from Polymaker Co., Ltd. (Suzhou, China). Poly (ethylene glycol) diacrylate (PEGDA; 600 g/mol, No. 1229220304) was provided by Ryoji Organic Chemical Co., Ltd. (Shanghai, China). Diphenyl (2,4,6-trimethylbenzoyl) phosphine oxide (DPPO; No. G2024033) was purchased from Shanghai Aladdin Biochemical Technology Co., Ltd. (Shanghai, China). Gelatin methacryloyl (GelMA; No. EFL-GM-90) and lithium phenyl (2,4,6-trimethylbenzoyl) phosphinate (LAP; No. EFL-LAP) were purchased from Yifuer Biotech Co., Ltd. (Hangzhou, China). Mouse fibroblast (L929) cells and bone marrow mesenchymal stem cells (BMSCs) were purchased from Shanghai Institutes for Biological Sciences (Shanghai, China). Roswell Park Memorial Institute (RPMI) 1640 medium, α-minimum essential medium (α-MEM; No. 2323107), fetal bovine serum (FBS; No. 2317362P), and penicillin/streptomycin were purchased from Thermo Fisher Scientific (Waltham, MA, USA). 4′,6-diamido-2-phenylindole dihydrochloride (DAPI; No. 20221024) solution and cell counting kit-8 (CCK-8; No. 907T014) were purchased from Beijing Solarbio Science & Technology Co., Ltd. (Beijing, China).

### 2.2. Preparation of Biomimetic Subchondral Bones by FDM

A 3D scanner (CR-Scan Lizard; Shenzhen Creality 3D Technology Co., Ltd., Shenzhen, China) was used to obtain subchondral bone models (Figure S1a), which were subsequently sliced using Cura 14.07 software (layer height = 0.15–0.30 mm, shell thickness = 0.8 mm, and fill density = 25%). Using PLA filament as the printing material, biomimetic subchondral bone specimens were prepared using an FDM printer (A3; JG AURORA, Shenzhen, China) at 190–220 °C and 30 mm/s.

Porous scaffold models with a fiber diameter of 0.4 mm (Figure S1b) were designed using SolidWorks 2016 software. The linear grid-filled porous scaffolds with side lengths of 0.2, 0.8, and 2.0 mm were named L-0.2, L-0.8, and L-2.0, respectively. The honeycomb grid-filled porous scaffolds with side lengths of 1.2, 1.6, and 2.0 mm were named H-1.2, H-1.6, and H-2.0, respectively. The models were sliced with a layer height of 0.2 mm, and porous scaffolds were FDM printed using PLA at 210 °C and 30 mm/s.

### 2.3. Preparation of Biomimetic Cartilages by DLP

Cartilage models (Figure S1c) were obtained by subtracting the subchondral bone models from osteochondral models. The models were sliced using NovaMaker v2.6.9 software with layer heights of 0.03–0.09 mm. The printing solution comprised 1% (*w*/*v*) DPPO mixed with PEGDA 600. The biomimetic cartilage specimens were printed using a DLP printer (NOVA3D BENE5; Shenzhen Nova Robot Technology Co., Ltd., Shenzhen, China) at 405 nm. The power rating is 20 W and the printing temperature is room temperature (approx. 25 °C). The exposure time was 20 s for the bottom three layers and 1–4 s for the other layers.

### 2.4. Preparation of Osteochondral Scaffolds

**Figure 1:**
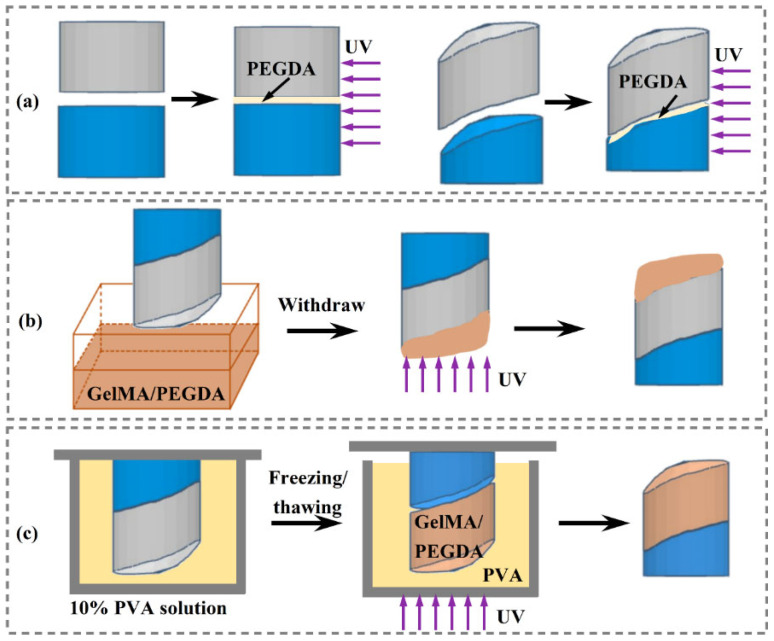
(**a**) Schematic of the preparation process for cylindrical and biomimetic scaffolds. Osteochondral scaffolds prepared using (**b**) the dip-coating method and (**c**) the casting method. GelMA, gelatin methacryloyl; PEGDA, poly (ethylene glycol) diacrylate; PVA, polyvinyl alcohol; UV, ultraviolet.

Cylindrical and biomimetic scaffolds with subchondral bone and cartilage layers were modeled (Figure S1d) and printed as described in Section 2.2 and Section 2.3 As shown in Figure 1a, PEGDA 600, containing 1% (*w*/*v*) DPPO, was added between the two layers and cured under ultraviolet (UV) light (365 nm, 18 W) for 60 s.

PEGDA displayed poor biocompatibility, and GelMA was added to improve the biocompatibility of the osteochondral scaffolds. To prepare the GelMA/PEGDA solution, 20% (*w*/*v*) GelMA was added to a solution containing 0.25% (*w*/*v*) LAP as a photoinitiator and mixed with PEDGA 600 at a ratio of 5:1. As shown in Figure 1b, osteochondral scaffolds with GelMA/PEGDA were prepared using the dip-coating method. The biomimetic scaffold in Figure 1a was dipped into the GelMA/PEGDA solution and cured under UV light for 60 s. The dip-coated osteochondral scaffold was obtained by performing the dip-coating procedure three times. As shown in Figure 1c, osteochondral scaffolds with GelMA/PEGDA were prepared using the casting method. The biomimetic scaffold in Figure 1a was fixed in a 10% polyvinyl alcohol (PVA) solution, and a PVA-negative mold was obtained using the freezing/thawing method. After removing the biomimetic scaffold, the GelMA/PEGDA solution was poured into the PVA-negative mold, followed by the addition of a biomimetic subchondral bone layer. The cast osteochondral scaffold was cured under UV light for 60 s.

### 2.5. Evaluation of Distance Deviation and Curvature Distributions

Digital morphologies of the printed subchondral bone and cartilage specimens were determined using a 3D scanning system [16] and used as test STL models. Using the printing STL models as reference models, the distance deviation was evaluated using Geomagic Qualify 2012 software through the best-fit alignment algorithm. Three-dimensional comparison maps were plotted, and deviation distributions were statistically analyzed. The relative frequency at a deviation range of −0.15 mm to 0.15 mm (*RF*_±0.15_) was determined as the maximum threshold for clinical use [23].

Based on the normal and in vivo defects in the distal femur, the normal group (G1) and defective group (G2) were established and prepared using FDM, as described in Section 2.2. Cylindrical and biomimetic scaffolds prepared in Section 2.4 were implanted into the defect site of G2 to obtain the cylindrical scaffold group (G3) and biomimetic scaffold group (G4), respectively. The digital morphologies of G1–G4 were obtained using a 3D scanner, and curvature distribution maps were obtained by cutting the bottom and refining the surface using Geomagic Wrap 2021.0.0 software. The digital morphologies of cartilage layers prepared by DLP, dip coating, and casting (Figure 1) were collected using a 3D scanner. The Gaussian curvature was measured using CloudCompare v2.13 software. Surface curvature distribution maps were plotted, and the relative frequency of the curvature distribution (*RF_c_*) was statistically analyzed.

### 2.6. Printing Fidelity and Porosity of Porous Scaffolds

The porous scaffolds were observed using a stereomicroscope (ZY-HD1400; Zongyuan Weiye, Shenzhen, China). The grid area of the porous scaffold (three grids per sample) was measured using Fiji 2.3.0 software. The printing fidelity of the porous scaffolds is defined as the ratio of the determined grid area to the designed grid area. The scaffold porosity was calculated using the gravimetric method [24] according to Equation (1), where *ρ_s_* and *ρ*_0_ are the densities of the porous and dense scaffolds, respectively. The lengths along the *x*-, *y*-, and *z*-axes were measured to calculate the volume.

(1) $$\mathrm{Porosity}(\%)=(1-\rho_{s}/\rho_{0})\;\times \;100$$

### 2.7. Evaluation of Mechanical Properties

A compression test [25] was performed using a universal testing machine (XZ-MNLF.10T; Zhejiang Yice Instrument Manufacturing Co., Ltd., Ningbo, China). Compression load–displacement (*F_c_*-*h_c_*) curves were recorded at a compression speed of 0.5 mm/min until a compressive force of 5 kN was achieved. Compression stress–strain (*σ_c_*-*ε_c_*) curves were plotted according to Equations (2) and (3), where *S* is the initial cross-sectional area and *L*_0_ is the initial height of the specimen. The compressive modulus (*E*) is the slope of the elastic region in the *σ_c_*-*ε_c_* curve, and Poisson’s ratio (*v*) is calculated according to the previous literature [26]. For porous scaffold specimens, the yield-point stress was recorded as the yield strength. The maximum stress of DLP-printed cylinder specimens was recorded as compressive strength.

(2) $$\sigma_{c}\;(\mathrm{MPa})=F_{c}/S$$

(3) $$\varepsilon_{c}(\%)\;=h_{c}/L_{0}\;\times \;100$$

Inspired by the evaluation of the self-healing ability of the hydrogel [27], a tensile test was performed to evaluate the bonding strength at the PLA-PEGDA interface of the osteochondral tensile sample. As shown in Figure S1e, the FDM-printed PLA and DLP-printed PEGDA samples were bonded using a UV-cured PEGDA 600 solution containing 1% (*w*/*v*) DPPO. Tensile samples with different interfacial curvatures were determined using a custom-made device (Figure S1e) by adding water at a constant rate of 3.6 mL/s, which was equivalent to loading a constant variable force of 0.035 N/s until the samples were fractured. The initial width (*W*_0_) and thickness (*T*_0_) of the sample were determined, and the ultimate tensile load (*F_t_*) was obtained to calculate the tensile stress at break (*σ_t_*) according to Equation (4). The bonding area was determined by multiplying the arc length of the interface with *T*_0_.

(4) $$\sigma_{t}\;(\mathrm{kPa})=F_{t}/(W_{0}\times T_{0})\;$$

### 2.8. Simulation and Validation of Biomechanical Behavior

FEA was used to investigate the internal biomechanical behavior of biomimetic knee joints using ANSYS 10.0 software. An interlayer with a biomimetic interface was inserted between the distal femoral and proximal tibial subchondral bones to mimic the cartilage and articular cavities. A solid 45 element with a size of 0.6 mm was used to mesh the models. *E* and *v* values of PLA and PEGDA, measured in Section 2.7 (*E* = 471 MPa, *v* = 0.30; *E* = 15.9 MPa, *v* = 0.35), were applied to the subchondral bones and interlayer, respectively. A static pressure of 0.103 MPa was applied to the surface of the distal femoral subchondral bone (approximately 380 mm^2^) of rabbits (4.0 kg). The first principal stress and total strain distributions were simulated. The compression deformation based on the deformed shapes was measured, and the *ε_c_* was calculated using Equation (3). The sagittal planes were acquired at X = −4, X = 0, and X = 4 to analyze the internal stress and strain distributions.

Subchondral bones and interlayers were printed using FDM and DLP, respectively. The compression test (described in Section 2.7) was conducted at a constant speed of 0.5 mm/min until the compressive force reached 100 N. Based on the *σ_c_*-*ε_c_* curve, the *ε_c_* corresponding to an *σ_c_* of 0.103 MPa was calculated to validate the FEA simulation.

Additionally, the biomechanical behavior of the biomimetic knee joints was simulated based on the models of G1–G4 in Section 2.5. The mechanical properties reported in the literature [28] (*E* = 19800 MPa, *v* = 0.30; *E* = 0.8 MPa, *v* = 0.40) were applied to the subchondral bones and interlayer, respectively. The mechanical properties of the PLA and PEGDA were applied to the subchondral bone and cartilage layers of the osteochondral scaffold, respectively. A static pressure of 0.103 MPa was applied, and the first principal stress and total strain distributions were simulated.

### 2.9. In Vitro Cell Adhesion and Proliferation Assays

For the cell adhesion assay, disk-shaped samples (14 mm diameter and 1 mm thickness) prepared using various materials were sterilized in a 24-well plate with 75% alcohol for 3 h and UV irradiation for 2 h. Briefly, L929 cell suspensions were evenly inoculated onto samples at a density of 2.5 × 10^5^ cells/well. RPMI 1640 medium containing 10% (*v*/*v*) FBS and 1% (*v*/*v*) penicillin/streptomycin was used to incubate L929 cells in a humidified 5% CO_2_ incubator at 37 °C. After incubation for 8 or 36 h, the adhered cells were collected from different samples and counted. The cell adhesion rate (*R_a_*) of the samples was calculated as the ratio of the number of adherent cells to the number of inoculated cells [29].

For the cell proliferation assay, disk-shaped samples (8 mm diameter and 1 mm thickness) prepared using various materials were sterilized in a 48-well plate with 75% alcohol for 3 h and UV irradiation for 2 h. BMSC suspensions were evenly inoculated onto samples at a density of 1.0 × 10^5^ cells/well, followed by incubation in α-MEM containing 10% (*v*/*v*) FBS and 1% (*v*/*v*) penicillin/streptomycin. To determine cell proliferation, samples were cultured for 1, 4, and 7 days and examined using a CCK-8 assay according to the manufacturer’s instructions. Briefly, 500 µL CCK-8 solution (CCK-8:α-MEM = 1:9) was added to each well. After incubation for 3 h, 100 µL of the medium was transferred to a 96-well plate. The optical density (OD) at 450 nm was measured using a microplate reader (Multiskan FC; Thermo Fisher Scientific Instrument Co., Ltd. Shanghai, China). After 4 days of culture, cell nuclei were stained with DAPI according to the manufacturer’s instructions, and cell nuclei were observed using a confocal laser scanning microscope (AX R; Nikon, Tokyo, Japan).

### 2.10. In Vivo Evaluation

All experimental procedures were conducted following the Guide for the Care and Use of Laboratory Animals at Zhejiang University of Technology (MGS20221207050) and the National Institutes of Health Guide for the Care and Use of Laboratory Animals (NIH Publications No. 85-23, revised 1996). A total of 27 male and female adult New Zealand white rabbits (2.0–2.5 kg) were divided into three groups: PEGDA scaffold, PVA scaffold, and osteochondral scaffold groups (9 rabbits per group). The rabbits were anesthetized with 25% ethyl carbamate (4 mL/kg), and the knee joint was exposed after dislocating the patella. An osteochondral defect (3 mm diameter and 4 mm depth) was created in the medial femoral condyle [30]. The defect in the left hind leg was left untreated as a control (untreated group), whereas different scaffolds were implanted into the defect site in the right hind leg. Finally, the operated knee joints were closed, and penicillin was administered intramuscularly once daily for 3 days to prevent infection. Histological samples of the distal femur were collected, examined, and photographed at 4, 8, and 12 weeks post-surgery. Histological sections of 5 µm thickness were stained with hematoxylin and eosin (H&E), safranin-O, and fast green (Saf-O/FG) to examine the regenerative zone. Histological samples obtained at 12 weeks post-surgery were further evaluated using the International Cartilage Repair Society (ICRS) macroscopic assessment scale for cartilage repair [29].

### 2.11. Statistical Analysis

Statistical analysis was performed using GraphPad Prism 6.0 software. Data are presented as mean ± standard deviation. Statistical significance is * *p* < 0.05, ** *p* < 0.01, *** *p* < 0.001, and **** *p* < 0.0001.

## 3. Results and Discussion

### 3.1. Morphologies and Internal Structure of Biomimetic Subchondral Bones

**Figure 2:**
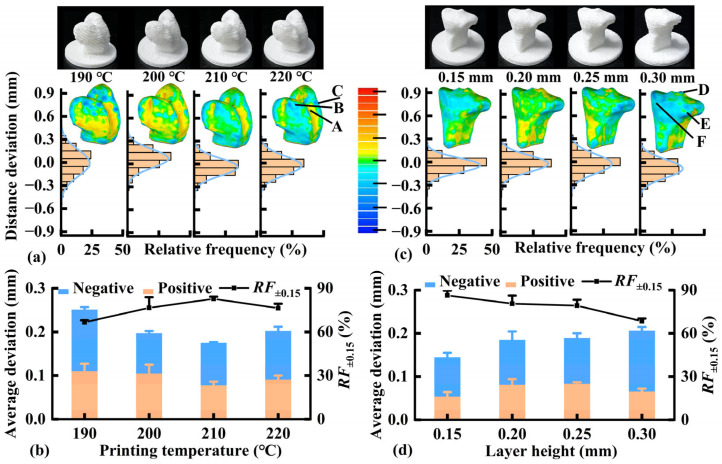
Effect of printing temperature on the distal femoral subchondral bone: (**a**) appearance, typical three-dimensional (3D) comparison map, and histogram of the deviation distribution; (**b**) average deviations and *RF*_±0.15_ (*n* = 3). Effect of layer height on the proximal tibial subchondral bone: (**c**) appearance, typical 3D comparison map, and histogram of the deviation distribution; (**d**) average deviations and *RF*_±0.15_ (*n* = 3). *RF*_±0.15_, the relative frequency at a deviation range of −0.15 mm to 0.15 mm.

Dimensional accuracy was evaluated using the distance deviation between the test and reference models. The influence of the FDM printing temperature on dimensional accuracy was assessed using the distal femoral subchondral bone as a model. As shown in Figure 2a, high temperatures of 210 and 220 °C resulted in smooth surfaces. A high printing temperature leads to good fusion among the layers, thereby reducing the surface roughness of the printed sample. Deviations were dispersed over a large range at low printing temperatures (190 and 200 °C). Conversely, the deviations were concentrated within a small range at high printing temperatures (210 and 220 °C). In the 3D comparison map, the positive deviations (marked in red) were mainly observed in the intercondylar area (zone A), whereas the negative deviations (marked in blue) were predominant in the medial and lateral condyles (zones B and C). As shown in Figure 2b, increasing the printing temperature from 190 to 210 °C decreased the average positive deviation from 0.110 to 0.078 mm and the average negative deviation from −0.141 to −0.097 mm, whereas *RF*_±0.15_ increased from 66.6% to 82.8%. Further increasing the printing temperature to 220 °C would increase the average deviations and decrease *RF*_±0.15_, which was related to the delayed solidification and deformation of the deposition layer. Accordingly, 210 °C was selected as the printing temperature for distal femoral subchondral bone printing.

Similarly, the effect of the FDM layer height on dimensional accuracy was evaluated using the proximal tibial subchondral bone as the model. The surface roughness of the printed specimens increased with increasing layer height (Figure 2c); this can be attributed to the stair-stepping effect owing to layer-by-layer printing. The deviations gradually dispersed over a larger range as the layer height increased. The positive deviations were mainly observed in the anterior intercondyle (zone D), whereas negative deviations primarily occurred in medial and lateral condyles (zones E and F). As shown in Figure 2d, increasing the layer height from 0.15 to 0.30 mm initially increased the average positive deviation from 0.054 to 0.084 mm, which subsequently decreased slightly to 0.066 mm. The average negative deviation increased from −0.091 to −0.140 mm, and *RF*_±0.15_ decreased from 86.3 to 68.4%; these findings can be attributed to the formation of interlayer gaps under a larger layer height, resulting in weakened interlayer bonds and inner stress that cause layer deformation. The observed result is consistent with that reported previously [31], where the length deviation increased with layer height increasing from 0.2 to 0.3 mm, and cracks and pores were formed at a layer height of 0.3 mm. The optimized layer height was 0.20 mm, considering its approximate dimensional accuracy but higher printing efficiency than the layer height of 0.15 mm.

**Figure 3:**
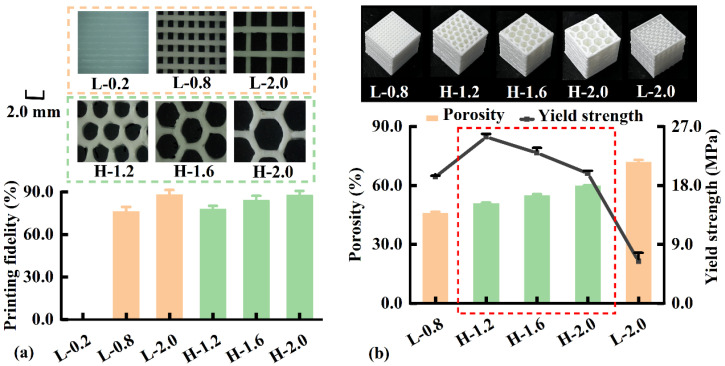
(**a**) Microstructure and printing fidelity (*n* = 3), (**b**) porosity and yield strength (*n* = 3) of the porous scaffolds.

Porous scaffolds with distinct filling patterns were printed to examine the internal structure of biomimetic subchondral bone. As shown in Figure 3a, when the side length of the linear grid was 0.2 mm (L-0.2), the grid was filled with the deformed PLA filament (no grid formation). For linear grids, the printing fidelity of the grid increased from 76.3% to 88.2% as the side length increased from 0.8 mm (L-0.8) to 2.0 mm (L-2.0). Considering honeycomb grids, the printing fidelity increased from 78.0% to 87.8% as the side length increased from 1.2 mm (H-1.2) to 2.0 mm (H-2.0). Honeycomb grids had a slightly lower printing fidelity than linear grids owing to the printing path overlap. The results revealed that FDM-printed porous scaffolds had a highly organized, linked porous structure. According to the literature [32], the porous scaffolds could be adjusted by the infill density. In the current study, the porous scaffolds could be adjusted by the filling patterns according to the biomimetic osteochondral scaffold requirements.

Porosity and mechanical properties are critical evaluation indicators of porous scaffolds, and the results are shown in Figure 3b. For linear grids, the porosity increased from 45.9% to 72.0%, whereas the yield strength decreased from 19.3 to 6.4 MPa as the side length increased from 0.8 mm (L-0.8) to 2.0 mm (L-2.0). For honeycomb grids, the porosity increased from 51.0% to 59.8%, whereas the yield strength decreased from 25.4 MPa to 19.8 MPa when the side length increased from 1.2 mm (H-1.2) to 2.0 mm (H-2.0). This result is consistent with that reported in a previous study [33], which suggested that the smaller the porosity, the greater the yield strength owing to the denser structure. The honeycomb grid scaffolds (H-1.2, H-1.6, and H-2.0) had higher porosities and yield strengths than the linear grid scaffold L-0.8. This can be attributed to the interconnected network of honeycomb units, which has a lower mass density and higher strength [34]. Thus, a honeycomb grid scaffold (H-1.2) with high porosity and yield strength was preferred for the internal structure of the biomimetic subchondral bone.

### 3.2. Morphologies of Biomimetic Cartilages

**Figure 4:**
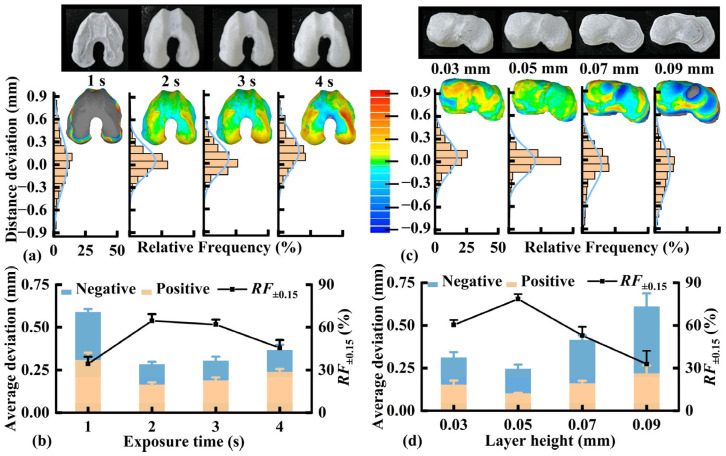
Effect of exposure time on the distal femoral cartilage: (**a**) appearance, typical three-dimensional (3D) comparison map, and histogram of the deviation distribution; (**b**) average deviations, *RF*_±0.15_ (*n* = 3), and effect of layer height on the proximal tibial cartilage; (**c**) appearance, typical 3D comparison map, and histogram of the deviation distribution; (**d**) average deviations and *RF*_±0.15_ (*n* = 3). *RF*_±0.15_, the relative frequency at a deviation range of −0.15 mm to 0.15 mm.

PEGDA has been widely used in tissue engineering owing to its mechanical stability and high printability. According to the result shown in Figure S2, PEGDA 600 was selected to prepare the biomimetic cartilage owing to its high compressive strength. The impact of DLP exposure time on dimensional accuracy was evaluated using the distal femoral cartilage as a model. As shown in Figure 4a, a short exposure time of 1 s resulted in an incomplete printed specimen due to a too-small polymerization depth. The majority of regions on the 3D comparison map were gray, revealing that the deviation exceeded the limit (10% of the total size of the specimen). Extending the exposure time from 2 to 4 s resulted in completely printed specimens. However, the red regions on the 3D comparison map were enlarged, suggesting a more positive deviation owing to over-curing. According to the histogram of the deviation distribution, the deviations were concentrated within a smaller range with a moderate exposure time of 2 or 3 s. As shown in Figure 4b, increasing the exposure time from 1 to 2 s decreased the average positive deviation from 0.309 to 0.165 mm and the average negative deviation from −0.280 to −0.118 mm while increasing *RF*_±0.15_ from 34.3% to 64.6%. However, further increasing the exposure time to 4 s increased the average deviations and decreased *RF*_±0.15_. These findings indicate that over-curing caused by prolonged exposure reduces the dimensional accuracy. Therefore, 2 s was established as the optimal exposure time for printing the biomimetic distal femoral cartilage.

Similarly, we evaluated the effect of DLP layer height on the dimensional accuracy of the proximal tibial cartilage. As shown in Figure 4c, increasing the layer height from 0.03 to 0.05 mm decreased the red regions (overprinted) on the 3D comparison map, and the deviations were concentrated in a smaller range. However, increasing the layer height from 0.07 to 0.09 mm resulted in incompletely printed specimens; the blue regions (insufficient printing) on the 3D comparison map were enlarged, and the deviation distribution was further dispersed. As shown in Figure 4d, increasing the layer height from 0.03 to 0.05 mm decreased the average positive deviation from 0.154 to 0.102 mm, reduced the average negative deviation from −0.158 to −0.145 mm, and increased *RF*_±0.15_ from 60.3% to 78.5%. However, further increasing the layer height to 0.09 mm increased the average deviations and decreased *RF*_±0.15_. An excessively low layer height causes excessive layer quantity [35] and over-curing owing to prolonged light scattering. However, excessive layer height leads to an incomplete formation owing to insufficient photocuring. Thus, 0.05 mm was considered the optimal layer height for biomimetic proximal tibial cartilage printing.

### 3.3. Bonding Strength and Curvature Distribution of Osteochondral Scaffolds

**Figure 5:**
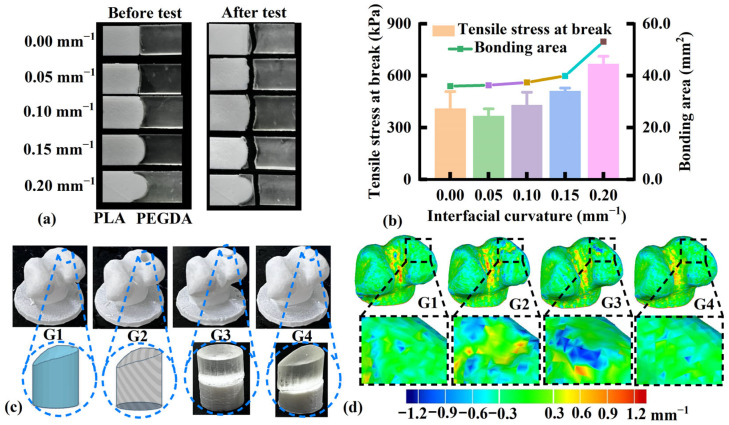
Influence of interfacial curvature on (**a**) osteochondral tensile samples before and after the tensile test; (**b**) tensile stress at break (*σ_t_*) (*n* = 3) and bonding area. (**c**) Appearance and (**d**) surface curvature distribution maps of distal femoral specimens in the normal group (G1), defective group (G2), cylindrical scaffold group (G3), and biomimetic scaffold group (G4).

The effect of PLA-PEGDA interface curvature on the bonding strength of osteochondral tensile samples is shown in Figure 5a,b. Without curvature (0.00 mm^−1^), the PLA-PEGDA interface was tightly bonded without a visible gap. Tensile fracture occurred at the PLA-PEGDA interface and PEGDA section with a *σ_t_* of 409.7 kPa. In the presence of a small curvature (0.05 mm^−1^), the printability of the PLA convex surface was poor, resulting in a discontinuous bonding interface. Therefore, tensile fracture occurred at the interface with a minimum *σ_t_* of 366.2 kPa. When increasing the curvature from 0.10 to 0.20 mm^−1^, the tensile fracture shifted from the interface to the PEGDA section, and the *σ_t_* increased from 430.4 to 667.6 kPa, which was associated with the expansion of the bonding area from 37.4 to 53.1 mm^2^. When further increasing the curvature to 0.20 mm^−1^, the *σ_t_* of osteochondral tensile samples exceeded the *σ_t_* of DLP-printed PEGDA samples, resulting in tensile fracture at the PEGDA section. Biomimetic interfacial curvature is deemed beneficial for ensuring the bonding strength of biomimetic osteochondral scaffolds.

Importantly, the surface morphology of the osteochondral scaffold must match that of the surrounding biological tissue. Based on the animal model of distal femur defects, cylindrical and biomimetic scaffolds were designed to evaluate changes in the surface curvature distribution before and after implantation. As shown in Figure 5c, the morphology of the biomimetic scaffold (G4) was better fitted to the defect site than that of the cylindrical scaffold (G3). As shown in Figure 5d, the defect site in G2 had an increased surface curvature in the medial femoral condyle (yellow and blue) when compared with the native tissue in G1. Following the implantation of a cylindrical scaffold (G3), the surface curvature at the defect site was further enlarged (blue and red areas). After implanting a biomimetic scaffold (G4), the surface curvature at the defect site returned to normal (similar to G1). Accordingly, biomimetic scaffolds were better matched to the surrounding biological tissue than traditional cylindrical scaffolds, ensuring safe and effective tissue regeneration.

### 3.4. Biomechanical Behavior Simulation and Validation

**Figure 6:**
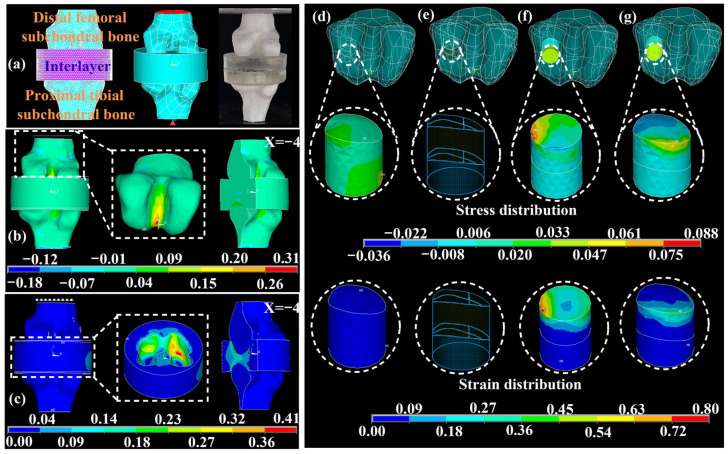
(**a**) Three-dimensional (3D) mesh model, a 3D pressure loaded model, and compression test of the knee joint; the simulated first principal (**b**) stress and (**c**) total strain distributions. Simulated first principal stress and strain distributions of (**d**) native tissue in G1, (**e**) defect site in G2, (**f**) scaffold in G3, and (**g**) scaffold in G4. G1, the normal group; G2, the defective group; G3, the cylindrical scaffold group; G4, the biomimetic scaffold group.

The biomechanical behavior of the biomimetic knee joint under gravity was simulated using the FEA method and validated using a compression test (Figure 6a). An interlayer (labeled in purple) was established between the distal femoral and proximal tibial subchondral bones to simulate the cartilage and articular cavity, enabling the simulation and validation of the biomechanical behavior of knee joints with irregular surfaces. A constant compression pressure of 0.103 MPa (red region) and a constraint in the XYZ direction (red triangle) were applied to the top and bottom surfaces, respectively. As shown in Figure 6b and Figure S3a, the intercondyle of the distal femoral subchondral bone withstood maximum stress (0.31 MPa), which was substantially lower than the yield strength of the H-1.2 scaffold (25.4 MPa) in Figure 3b. The stress distribution in the sagittal plane at X = −4 was uniform, and the stress was transferred from the distal femoral subchondral bone to the interlayer, followed by the proximal tibial subchondral bone, avoiding stress shielding [36]. Based on the observed results, the biomimetic subchondral bone exhibited good compression resistance. As shown in Figure 6c and Figure S3b, the lateral condyles of the distal femur in the interlayer withstood the maximum total strain, revealing the cushioning effect of the cartilage and articular cavity. Comparing the simulation results with the initial undeformed model (marked with a white dashed line), the simulated compression deformation was 0.65 mm, equivalent to 2.03% of the simulated *ε_c_*. This finding was consistent with the compression test results presented in Figure S3c, where the *ε_c_* was 1.77 ± 0.16% under a *σ_c_* of 0.103 MPa. Based on the biomimetic knee joint, a visual simulation of the biomechanical behavior can be used to select appropriate scaffolds, thereby considerably reducing the need for animal experiments.

The biomechanical behavior of the osteochondral scaffolds was simulated based on the biomimetic knee joint FEA model described above. The stress and strain distributions of the native tissue, defect site, and scaffolds were extracted and shown in Figure 6d–g. The cylindrical scaffold in G3 exhibited larger and more unevenly distributed stress and strain than the native tissue in G1. The lateral surface of the cartilage layer in the cylindrical scaffold withstood the ultimate stress and strain (0.088 MPa and 0.80%, respectively). Conversely, the biomimetic scaffold in G4 had a smaller and more uniformly distributed stress and strain than the cylindrical scaffold in G3, although they were comparable to those of the native tissue in G1. Accordingly, biomimetic morphology could effectively reduce the stress and strain of the scaffold, thereby facilitating its in vivo stability.

### 3.5. Cytocompatibility of the Osteochondral Scaffold

**Figure 7:**
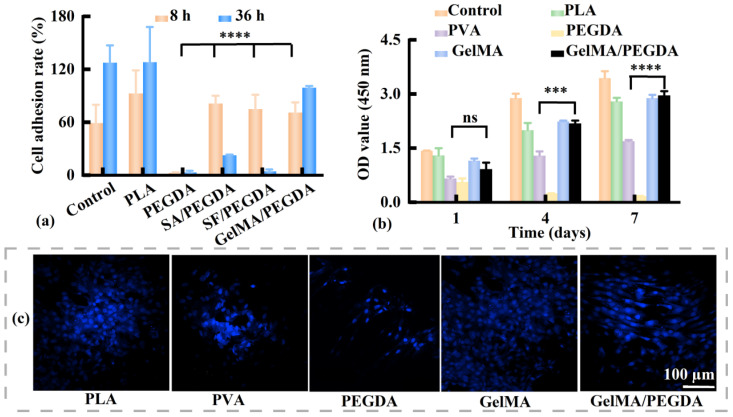
Influence of material on (**a**) the cell adhesion rate (*R_a_*) of L929 cells (*n* = 6) and (**b**) OD value of BMSCs cultured for 1, 4, and 7 days (*n* = 6). (**c**) DAPI staining of the nuclei in the BMSCs cultured for 4 days. L929, mouse fibroblasts; OD, optical density; BMSCs, bone marrow mesenchymal stem cells; DAPI, 4′,6-diamido-2-phenylindole dihydrochloride; PLA, polylactic acid; PEGDA, poly (ethylene glycol) diacrylate; SA, sodium alginate; SF, silk fibroin; GelMA, gelatin methacryloyl; PVA, polyvinyl alcohol. ns: no significance, *** *p* < 0.001, **** *p* < 0.0001.

The attachment of L929 cells to the materials was used to evaluate cytotoxicity. The *R_a_* values of L929 cells on various osteochondral scaffold materials are shown in Figure 7a. PLA is an FDA-approved polymer for orthopedic implants [37], and the *R_a_* values of PLA (92.7% and 128.1% at 8 and 36 h, respectively) were higher than those of the control group. PEGDA is another widely used polymer in tissue engineering owing to its mechanical stability and high printability [38]. However, the *R_a_* value of PEGDA was substantially low (3.3% at 36 h). The addition of biocompatible polymers sodium alginate (SA), silk fibroin (SF), and GelMA to PEGDA significantly increased the *R_a_* value to 81.4%, 75.0%, and 71.0% at 8 h, respectively (*p* < 0.0001). Compared with the significantly reduced *R_a_* value of SA/PEGDA and SF/PEGDA (22.7% and 4.5%, respectively) at 36 h, the *R_a_* value of GelMA/PEGDA at 36 h further increased to 99.2%. This finding indicates that PLA and GelMA/PEGDA exhibit favorable biocompatibility and are promising candidates for the subchondral bone and cartilage layers of osteochondral scaffolds.

The BMSCs were used for the biocompatibility evaluation. Using FDA-approved PVA [39] as a control material, we compared the proliferation of BMSCs on different materials to preliminarily evaluate their osteochondral repair capacity (Figure 7b). Except for PEGDA, the OD values of all groups continued increasing during the 7-day culture period, revealing cell proliferation. GelMA and GelMA/PEGDA had higher OD values than those of PVA, indicating the superior cartilage regeneration potential of GelMA/PEGDA. As shown in Figure 7c, a large number of BMSC nuclei were observed in the commonly used PLA and PVA after culturing for 4 days. More cells were observed on GelMA and GelMA/PEGDA than on PEGDA. PEGDA displayed high mechanical strength but poor biocompatibility, whereas GelMA displayed good biocompatibility but brittleness [40]. The GelMA/PEGDA complex could provide a favorable microenvironment for cell proliferation and ensure adequate mechanical strength.

### 3.6. Surface Curvature of the Osteochondral Scaffold

**Figure 8:**
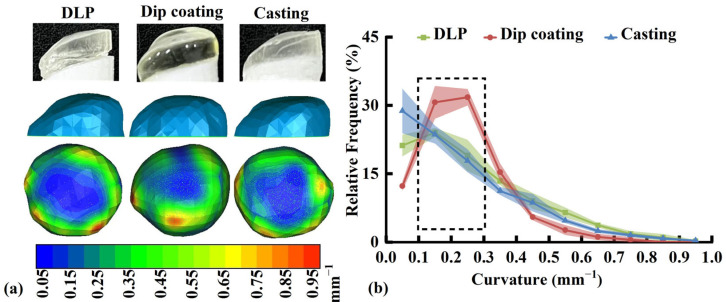
Cartilage layer prepared using the DLP, dip-coating, and casting methods: (**a**) appearance, digital models, surface curvature distribution maps, and (**b**) curvature distribution curves (*n* = 3). DLP, digital light processing.

Owing to the poor printability of GelMA/PEGDA below 50 °C, the cartilage layer was prepared using the dip-coating and casting methods. A DLP-printed cartilage layer based on PEGDA was used as the control, and the effects of different methods on surface curvature distribution are shown in Figure 8a. Compared with the DLP-printed specimens, the dip-coated specimens had a smooth edge, resulting in an increased curvature (enlarged green area) at the edges in the surface curvature distribution map. Conversely, cast specimens had a similar appearance and surface curvature map to those of DLP-printed specimens. As shown in Figure 8b, among curvature ranges of 0.1–0.2 and 0.2–0.3 mm^−1^ (black dashed frame), the *RFc* of the dip-coated specimens (30.7% and 31.8%, respectively) were higher than those of cast specimens (23.6% and 17.8%, respectively), which were similar to those of the DLP-printed specimens (24.1% and 19.0%, respectively). The Pearson correlation coefficient between curves of the cast and DLP-printed specimens (0.967) was higher than that between curves of the dip-coated and DLP-printed specimens (0.890). This finding indicates that the curvature distribution of the cast specimen (blue curve) closely approximates that of the DLP-printed specimen (green curve). Thus, the casting method is preferred for preparing the cartilage layer of the osteochondral scaffold to ensure a biomimetic curvature.

### 3.7. Tissue Repair and Osteochondral Regeneration of Osteochondral Scaffold

**Figure 9:**
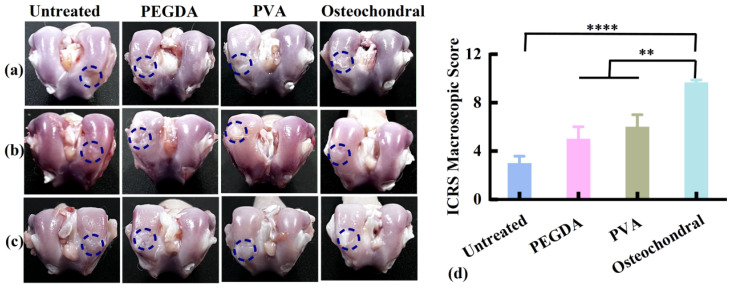
Appearance of the distal femoral histological samples at (**a**) 4, (**b**) 8, and (**c**) 12 weeks post-surgery. The blue circle indicates the location of defect creation. (**d**) ICRS macroscopic score for cartilage regeneration at 12 weeks post-surgery (*n* = 3). ICRS, International Cartilage Repair Society; PEGDA, poly (ethylene glycol) diacrylate; PVA, polyvinyl alcohol. ** *p* < 0.01, **** *p* < 0.0001.

Based on the results shown in Figure 8, the osteochondral scaffold prepared using the casting method was further used to examine tissue repair. The in vivo osteochondral regeneration potential of scaffolds was examined in rabbits with full-thickness osteochondral defects. At 4 weeks post-surgery (Figure 9a), substantial surface defects persisted in the distal femoral histological samples of the PEGDA and PVA scaffold groups and the untreated group. Conversely, no evident defects were observed in the osteochondral scaffold group. At 8 and 12 weeks post-surgery (Figure 9b,c), regenerated tissues were visible in all groups. The regenerated tissue of the osteochondral scaffold group exhibited better fusion with the surrounding cartilage than that of the PEGDA and PVA scaffold groups, resulting in a smoother surface. Furthermore, the ICRS macroscopic score of regenerated tissues was assessed at 12 weeks post-surgery (Figure 9d). The cartilage of the untreated group was severely abnormal (grade IV), with a low score of 3.0. The cartilage of the PEGDA and PVA scaffold groups was abnormal (grade III), presenting scores of 5.0 and 6.0, respectively. The osteochondral scaffold group had nearly normal (Grade II) cartilage, with a high score of 9.7.

**Figure 10:**
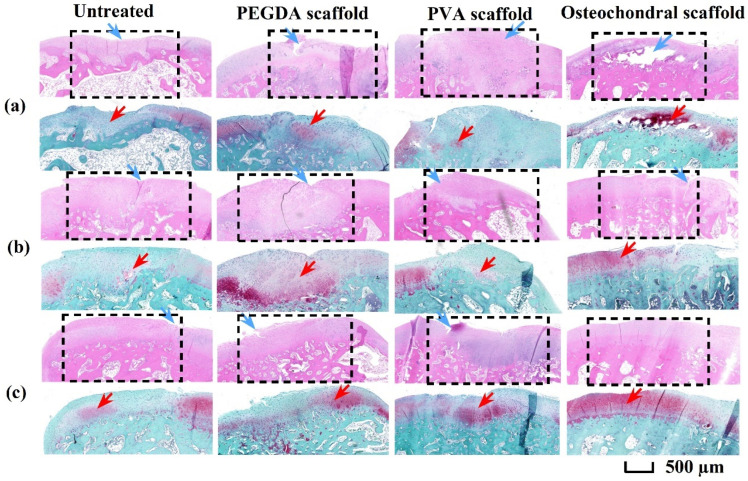
H&E and Saf-O/FG staining of osteochondral tissues in different groups at (**a**) 4, (**b**) 8, and (**c**) 12 weeks post-surgery. Blue and red arrows indicate the surface defect and chondrocytes in the regenerative zone, respectively. The black dashed frame indicates the regenerative zone. H&E, hematoxylin–eosin; Saf-O/FG, safranin-O and fast green; PEGDA, poly (ethylene glycol) diacrylate; PVA, polyvinyl alcohol.

The structural integrity of the regenerative zone (black dashed frame) was evaluated using H&E staining, whereas chondrocyte regeneration and distribution were evaluated using Saf-O/FG staining. Four weeks post-surgery (Figure 10a), the interior cavity (white region) and surface defects (blue arrow) were visible in the untreated group, indicating incomplete tissue regeneration. The regenerative zone exhibited weak Saf-O staining (red arrow), suggesting the presence of a limited number of new chondrocytes. In the PEGDA and PVA scaffold groups, most surface defects were repaired, but a few chondrocytes were observed in the regenerative zone. Considering the osteochondral scaffold group, the surface defect was completely repaired, and robust Saf-O staining was observed, indicating numerous mature chondrocytes. At 8 weeks post-surgery (Figure 10b), the defects in all groups were filled with regenerated tissues. However, only a small number of chondrocytes were regenerated in the untreated and PVA scaffold groups. The PEGDA scaffold group had a high number of regenerated chondrocytes, whereas the osteochondral scaffold group had more uniformly regenerated chondrocytes. In all groups, surface defects were repaired 12 weeks post-surgery (Figure 10c). Numerous mature chondrocytes were observed in the osteochondral scaffold group, suggesting the emergence of an abundant cartilage matrix in the regenerative region. The osteochondral scaffold displayed the best osteochondral regeneration ability, which was associated with the high biocompatibility of GelMA/PEGDA in the cartilage layer, as well as the biomimetic dual-layer structure that facilitated nutrient transport and mechanical support.

## 4. Conclusions

In the present study, reverse engineering was employed to establish biomimetic models of subchondral bones and cartilage. Subchondral bones with biomimetic morphology and an internal structure were prepared using FDM, whereas cartilage with biomimetic morphology and mechanical properties were prepared using DLP. The results revealed that the interfacial curvature of the biomimetic osteochondral scaffolds was beneficial for bonding strength. Importantly, the biomimetic scaffolds exhibited curvature distribution and biomechanical behavior more consistent with native tissues than with traditional cylindrical scaffolds. A GelMA/PEGDA cartilage layer was prepared using the casting method to achieve high biocompatibility and biomimetic morphology. The optimized osteochondral scaffold could repair osteochondral injury in rabbits faster and better than traditional scaffolds. In this study, biodegradable scaffolds were prepared using biodegradable materials, such as PLA, PEGDA, and GelMA. The degradation of PLA in vivo may take a few years [37], while the degradation of PEGDA and GelMA in vivo may take a few months [41]. Therefore, their degradation behavior and long-term safety should be further studied. Additionally, larger defects (critical defects) in large animals should be used to evaluate the in vivo osteochondral regeneration potential of scaffolds.

Biomimetic osteochondral scaffolds prepared based on reverse engineering combined with 3D printing technologies not only promote the treatment of osteochondral injuries in knee joints but could facilitate the treatment of osteochondral injuries in other joints (i.e., shoulder, elbow, hip, and ankle joints). Moreover, biological factors (i.e., growth factors and cells) and physical factors (i.e., mechanical force, electrical stimulation, and magnetic stimulation) can be combined with osteochondral scaffolds to promote tissue regeneration.

## Data Availability

Dataset available on request from the authors.

## Supplementary Materials

The following supporting information can be downloaded at https://www.mdpi.com/article/10.3390/pharmaceutics17020153/s1, Figure S1: Models of (a) subchondral bones, (b) porous scaffolds, and (c) cartilages. (d) Models of cylindrical and biomimetic scaffolds, and (e) osteochondral tensile sample for tensile test. PEGDA, poly (ethylene glycol) diacrylate; PLA, polylactic acid. Figure S2: (a) Typical compression stress-strain (σc-εc) curve and (b) compressive strength (*n* = 3) of PEGDA with different molecular weights. PEGDA, poly (ethylene glycol) diacrylate. Figure S3: The simulated first principal (a) stress and (b) total strain distributions of the sagittal planes at different positions; (c) compression result of the knee joint.

## Nomenclature

3D	three dimensional	*F_c_*	compression load
FDM	fused deposition modeling	*h_c_*	compression displacement
DLP	digital light processing	*σ_c_*	compression stress
FEA	finite element analysis	*ε_c_*	compression strain
PLA	polylactic acid	*S*	initial cross-section area
PEGDA	poly (ethylene glycol) diacrylate	*L*_0_	initial height
DPPO	diphenyl (2,4,6-trimethylbenzoyl) phosphine oxide	*E*	compressive modulus
GelMA	gelatin methacryloyl	*v*	Poisson’s ratio
LAP	lithium phenyl (2,4,6-trimethylbenzoyl) phosphinate	*W*_0_	initial width
L929	mouse fibroblast	*T*_0_	initial thickness
BMSCs	bone marrow mesenchymal stem cells	*F_t_*	ultimate tensile load
RPMI	Roswell Park Memorial Institute	*σ_t_*	tensile stress at break
α-MEM	α-minimum essential medium	*R_a_*	cell adhesion rate
FBS	fetal bovine serum	OD	optical density
DAPI	4′,6-diamido-2-phenylindole dihydrochloride	PVA	polyvinyl alcohol
CCK-8	cell counting kit-8	H&E	hematoxylin–eosin
UV	ultraviolet	Saf-O/FG	safranin-O and fast green
*RF*_±0.15_	relative frequency when the deviation ranges from −0.15 mm to 0.15 mm	ICRS	International Cartilage Repair Society
*RF_c_*	relative frequency of curvature distribution	SA	sodium alginate
*ρ_s_*	density of the porous scaffold	SF	silk fibroin
*ρ*_0_	density of the dense scaffold		
