# Supplementary material for: Three-Dimensional-Printed Osteochondral Scaffold with Biomimetic Surface Curvature for Osteochondral Regeneration
Source: Pharmaceutics. 2025 Jan 23;17(2):153. doi: 10.3390/pharmaceutics17020153 (PMC11859329; doi:10.3390/pharmaceutics17020153)
Supplement: Supplementary file 1 [file pharmaceutics-17-00153-s001.zip › pharmaceutics-3380550-supplementary.pdf]

Supporting Information

S1. Printing models

Distal femoral and proximal tibial subchondral bone models were constructed using a three-dimensional (3D) scanner with the CR Studio software. The models were smoothed and cropped using the Geomagic Wrap software, and a bottom layer was added for printing (Figure S1a). Dimensions of the porous scaffolds are shown in Figure S1b. The printing models of cartilages are shown in Figure S1c. Additionally, schematic diagrams illustrating the preparation process of the osteochondral tensile sample and the custom-made device are presented in Figure S1d. Finally, the dimensions of the subchondral bone and cartilage layers in cylindrical and biomimetic scaffolds are labeled in Figure S1e.

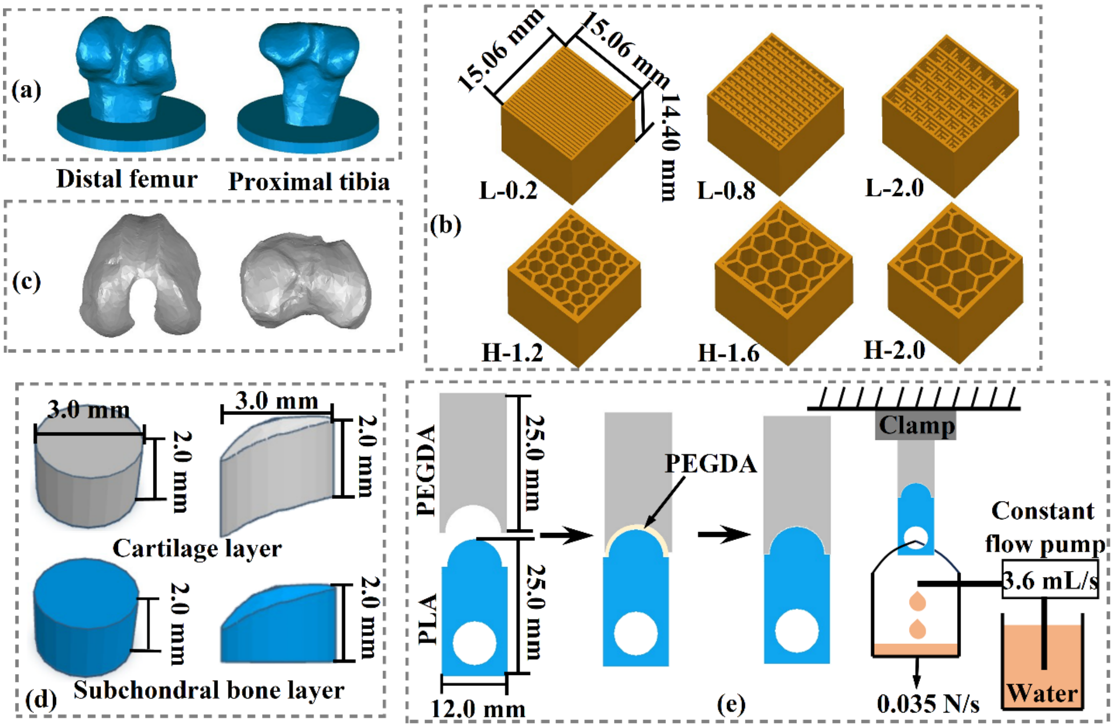

Figure S1 Models of (a) subchondral bones, (b) porous scaffolds, and (c) cartilages. (d) Models of cylindrical and biomimetic scaffolds, and (e) osteochondral tensile sample for tensile test. PEGDA, poly (ethylene glycol) diacrylate; PLA, polylactic acid.

S2. Mechanical properties of PEGDA

A cylindrical model with a diameter of 8 mm and height of 5 mm was designed

using SolidWorks 2016 software. The model was sliced with a layer height of 0.05 mm. The printing solution comprised 1% (w/v) diphenyl (2,4,6-trimethylbenzoyl) phosphine oxide mixed with PEGDA (400, 600, and 1000). Compression specimens were prepared using a digital light processing (DLP) printer. The exposure time was 20 s for the bottom three layers and 2 s for the other layers.

Figure S2a presents compression stress-strain (σ_c-ε_c) curves of DLP-printed cylinders using PEGDA with different molecular weights. All specimens exhibited brittle behavior with a linear elastic region until the applied stress reached compressive strength, at which point the specimens cracked, as evidenced by an abrupt drop in stress to zero. Figure S2b presents the effect of the PEGDA molecular weight on the compressive strength of the DLP-printed specimens. Increasing the molecular weight from 400 to 600 g/mol enhanced the compressive strength from 16.3 to 28.1 MPa; this finding can be attributed to the crosslinking of long PEGDA chains, which results in a robust network. Nevertheless, the compressive strength decreased to 22.5 MPa as the molecular weight was further increased to 1000 g/mol; this can be explained by the crosslinking of substantially long PEGDA chains, resulting in a looser network.

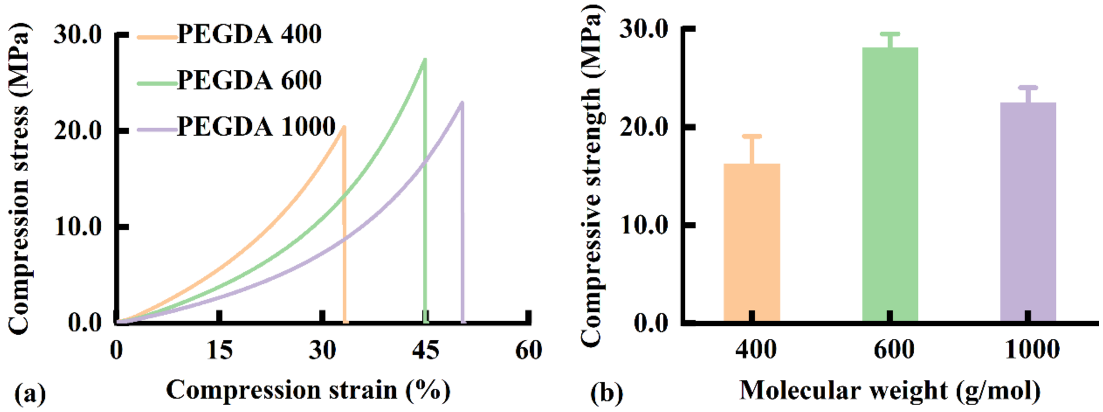

Figure S2 (a) Typical compression stress-strain (σ_c-ε_c) curve and (b) compressive strength (n = 3) of PEGDA with different molecular weights. PEGDA, poly (ethylene glycol) diacrylate.

S3. Biomechanical behavior of the knee joint

For the knee joint, biomechanical behavior simulation and validation were conducted. The stress distribution in the sagittal planes at X = −4 and X = 4 was uniform (Figure S3a). The stress in the sagittal plane at X = 0 was concentrated in the intercondylar portion of the distal femoral subchondral bone (white dashed circle). The

total strain in all sagittal planes (Figure S3b) was concentrated in the interlayer, indicating that the cartilage and articular cavity displayed more notable deformations than the subchondral bones in the weight-bearing condition. The compression result of the knee joint was shown in Figure S3c, and the ε_c was 1.77% ± 0.16% under σ_c of 0.103 MPa.

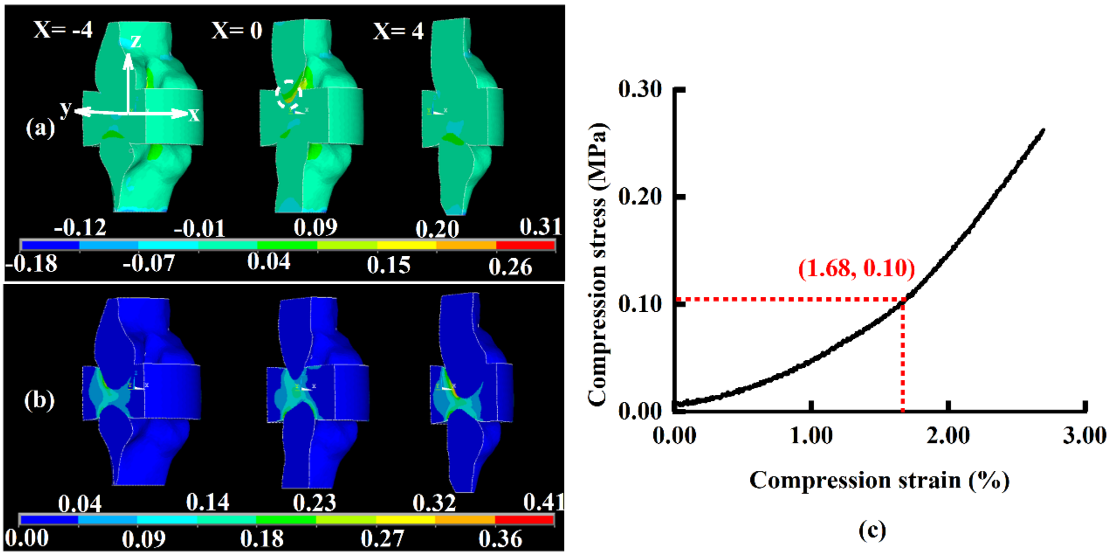

Figure S3 The simulated first principal (a) stress and (b) total strain distributions of the sagittal planes at different positions; (c) compression result of the knee joint.
